# A novel nanomicelle composed from PEGylated TB di-peptide could be successfully used as a BCG booster

**DOI:** 10.22038/IJBMS.2022.61373.13583

**Published:** 2022-02

**Authors:** Zohreh Firouzi, Mahmoud Reza Jaafari, Mojtaba Sankian, Sirwan Zare, Mohsen Tafaghodi

**Affiliations:** 1 Department of Pharmaceutical Nanotechnology, School of Pharmacy, Mashhad University of Medical Sciences, Mashhad, Iran; 2 Student Research Committee, Mashhad University of Medical Sciences, Mashhad, Iran; 3 Nanotechnology Research Center, Pharmaceutical Technology Institute, Mashhad University of Medical Sciences, Mashhad, Iran; 4 Immunobiochemistry Laboratory, Immunology Research Center, School of Medicine, Mashhad University of Medical Sciences, Mashhad, Iran

**Keywords:** Nanovaccine, Nasal and parenteral – immunization, Recombinant fusion – peptide, Self-assembled – nanomicelles, Tuberculosis

## Abstract

**Objective(s):**

Tuberculosis affects one-third of the world’s population and leads to a high rate of morbidity and mortality. Bacillus Chalmette–Guerin (BCG) as the only approved vaccine for the *Mycobacterium tuberculosis *(*Mtb*) does not show enough protection in the vaccinated population.

**Materials and Methods:**

The main aim of this study was to prepare a self-assembled nanomicelle composed from a di-block polymer in which, a di-fusion peptide was the hydrophobic block and polyethylene glycol (PEG) was the hydrophilic block. The micelles were characterized *in vitro* and *in vivo* as an antigen delivery system/adjuvant both with and without a prime BCG.

**Results:**

The micellar nanovaccine was able to elicit good dendritic cell maturation. Nanomicelles could efficiently induce systemic cytokines as well as nasal secretory predominant antibody titers (sIgA). The expression pattern of cytokines indicated the superiority of cellular immunity. Nasal administration of two doses of nanomicelles after a prime subcutaneous administration of BCG induced the highest mucosal and systemic immune responses.

**Conclusion:**

Based on our results PEG-HspX/EsxS self-assembled nanomicelle is highly immunogenic and can be considered a potential vaccine candidate against *Mtb* to boost BCG efficiency.

## Introduction

*Mycobacterium tuberculosis* (*Mtb*) mainly attacks the host respiratory system and is transmitted by aerosol from one host to another (1). According to the World Health Organization (WHO), TB affects one-third of the world population and exhibits high morbidity and mortality rates. A developed type of TB known as “*latent tuberculosis*” hinders the process of tuberculosis treatment and unfortunately in about 25% of the infected population has hidden symptoms. *Latent tuberculosis* can also spread in the hidden state (2).

Despite the development of therapeutic agents, today Bacillus Chalmette-Guerin (BCG) is the only TB vaccine that is clinically used widely (3). One challenge in vaccination against *Mtb* is the fact that the BCG vaccine has not shown enough efficacy and varies within different ethnic communities. It is experimentally shown that the induced protectively can vary from 0% to 80% in different cases. On the other hand, BCG exhibits almost no prevention against the reactivation of the dormant bacilli (4). Consequently, it is necessary today to develop a new effective vaccine against TB with more stable performance and higher level of protection. This challenge is addressed as an important and open research question in the literature (3). Generally, two approaches can be followed: the first is to completely substitute the current BCG with a newly designed vaccine, and the second is to utilize the boosting mechanism by combinational administration of BCG and a new vaccine. The second approach is addressed as a more effective method in the literature (5). In this research study, we have practically demonstrated the viability of the second approach (boosting method) in improving the immune responses. We have utilized the Latency-Associated Antigens (LAA) as subunit vaccines which contain one or a few purified sections of the dominant *Mtb* antigens. This approach is addressed as a growing trend in TB vaccination in order to boost immune responses (5). It is reported that LAA can give rise to a stronger CD4^+^ T helper1 (Th1)-like immune response for both active and latent infections (6, 7). In other words, LAA causes CD4+ T cell proliferation and a better expression of the pro-inflammatory Th1-type cytokines e.g., interleukin-12 (IL-12), interferon-gamma (IFN-γ), and tumor necrosis factor (TNF-α) (6-8).

The recombinant antigenic peptides are safe, but they are weak in terms of immunogenicity. One of the most dominant *Mtb* antigen genes is HspX which shows good capability in stimulating the Th1-like immune responses. Experimental evidence shows that HspX induces the IFN-γ production in T cells and induces stronger Th1-like immune response (9). In addition to that, T cells expose specific receptors for EsxS proteins which are reported as relatively good stimulators of the cellular immune system (10). As a result, EsxS protein can be also considered as another suitable candidate for a TB vaccine. However, most of these subunit vaccines have not represented significant immunogenicity and thereby must be reformulated and combined by immunoadjuvants (11). 

In order to increase the immune responses against the loaded/attached antigens, the nanoparticulate vaccine delivery systems/adjuvants could be used (12, 13). It is practically shown that HspX-EsxS fusion protein as a vaccine candidate can elicit stronger Th1 and Th2 responses when it is encapsulated with nanoparticles such as PLGA (14). 

Nanomicelles as a subset of nanoparticles can be considered as potent carriers for the antigen delivery system. Micelles provide multiple advantages: the flexible nature of the micelles’ segments and their narrow size distribution, low energy required for self-assembly, the ability to carry a wide range of therapeutic agents and antigens, and the possibility to decorate the surface of the nanomicelles for targeted delivery (15). 

In the present study a di-block composed of a hydrophilic block, polyethylene glycol, and HspX-EsxS di-fusion protein as hydrophobic block, were synthesized. This amphiphilic di-block can be self-assembled into nanomicelles in which the di-fusion protein as hydrophobic block is placed into the core of the nanomicelle. The physiochemical properties and the stability of this constructed self-assembled nanomicelle are studied and its immunogenicity is evaluated both *ex vivo *and *in vivo.*

## Materials and Methods

***Materials***

Polyethylene glycol (6000 g/mol) was purchased from Scharlau (Spain). N-(3-dimethylaminopropyl)-N′-ethyl carbodiimide hydrochloride (EDC) and N- hydroxysuccinimide (NHS) were purchased from Sigma-Aldrich (USA). RPMI 1640 medium and fetal calf serum (FCS) were purchased from Gibco (USA). IMDM media was purchased from Sigma-Aldrich (USA). Lipopolysaccharide (LPS), mouse IL-4, and recombinant mouse granulocyte-macrophage colony-stimulating factor (GM-CSF) were purchased from Biolegend Systems (Minneapolis, MN, USA). Fluorochrome-labeled anti-mouse monoclonal antibodies (FITC-CD11c, PE-CD40, PE-CD80, PE-CD86, PE Rat anti-Mouse I-Ad/I-Ed) were purchased from BD Biosciences. Horseradish peroxidase-conjugated goat anti-mouse IgA was obtained from Southern Biotechnologies (Birmingham, AL, USA). Mouse IL-4, IL-17, TGF-beta, and IFN-γ ELISA kits were purchased from Biolegend (San Diego, CA, USA). Ni-NTA affinity chromatography kit was purchased from QIAGEN (USA). All other reagents were of analytical grade.

***Construction, expression, and purification of HspX-EsxS fusion protein***

HspX-EsxS di-fusion protein was expressed as described in the previous study (16). Briefly, the optimized gene sequence of the recombinant di-fusion protein was designed. The corresponding protein was expressed in the bacterial host, extracted by lysing of the cells and purified by Ni-NTA His affinity chromatography, and evaluated by SDS-PAGE analysis and BCA protein assay kit.

***Preparation and characterization of self-assembled nanostructure***

*Synthesis of carboxylated polyethylene glycol*

In order to synthesize the di-block copolymer, polyethylene glycol (MW~6,000 Da) was conjugated to HspX-EsxS di-fusion protein. Briefly, the end hydroxyl group of polyethylene glycol was first converted to the carboxyl group. For this purpose, 5 mmol of polyethylene glycol along with 10 mmol of maleic anhydride was dissolved in 10 ml of toluene and placed in a reflux system at 70 °C and a paraffin bath for 24 hr. After the reaction, the contents were washed with diethyl ether and the resultant precipitate was dried for one day at room temperature (17).

*Synthesis of PEGylated-protein*

For conjugation, 50 mg of carboxy-PEG was dissolved in 10 ml pure double distilled water and the solution was stirred for 10 min at 40 °C. 1-ethyl-3-(3-dimethylaminopropyl)-carbodiimide (1:10 carboxy PEG:EDC molar ratio) and N-hydroxysuccinimide (1:10 carboxy PEG:NHS molar ratio) was added and the mixture was stirred at room temperature for 1 hr until activation of the carboxyl group for direct amide coupling reaction (18, 19). The protein solution with a molar ratio of 1:2 was added dropwise to the polymer solution and the solution was allowed to stand for 24 hr at 4 °C. After 24 hr, the solution was purified with a dialysis bag with a 14 kDa molecular weight cut-off (Spectrum Laboratories, Inc., Rancho Dominquez, CA, USA). Ultimately, the dialysate was freeze-dried to yield the final copolymer and kept at −20 °C until future use. Verification of protein PEGylating was conducted by FTIR spectroscopy (FTIR, PerkinElmer Inc, USA) and ^1^HNMR spectra using Bruker 300 MHz nuclear magnetic resonance instrument. In brief, a stock solution of copolymer in DMSO (20 mg/ml) was prepared and filtered to obtain a clear solution. Then, 200 μl of this solution was used for ^1^HNMR spectroscopy. For the FTIR spectroscopy, a mixture of copolymer and KBr salt (1% copolymer in KBr) was prepared. A pellet from KBr and copolymer mixture was prepared and applied in FTIR infrared spectroscopy to be analyzed.

*Determination of critical micelle concentration (CMC)*

**Figure 1:**
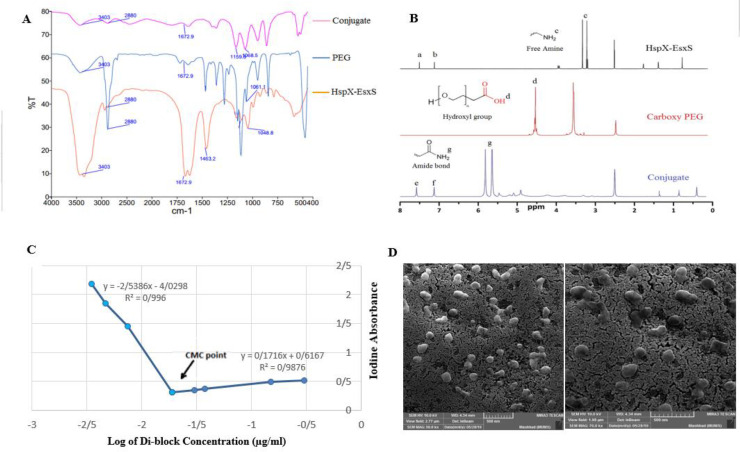
(A) FT-IR spectrum of Hspx-EsxS and FT-IR spectrum of Polyethylene Glycol / HspX-EsxS di-block (Conjugate), (B) the comparison of 1H-NMR spectrum in HspX-EsxS, Polyethylene Glycol and Conjugate; a,b,e and f are associated with aromatic amino acids in HspX-EsxS, (C) Determination of critical micelle concentration (CMC) for the nanomicelle (Polyethylene Glycol - fusion protein), (D) Scanning Electron Microscopy (SEM) images for the Polyethylene Glycol - protein self-assembled nanoparticles

To ensure micelle formation and assess the maximum concentration required for its formation in an aqueous medium, CMC was determined with a dye solubilization technique with minor modifications (20). In brief, serially diluted suspensions of copolymer (0.001–5 mg/ml) were prepared in distilled water. Iodine was used as a hydrophobic probe (5 μl from 8 mg iodine /ml acetone). Solutions were kept overnight at room temperature to reach equilibrium and finally, the absorption spectra of samples were measured at 369 nm by UV–Vis spectra (UV-160 A Shimadzu, Japan). The log concentration against absorption was plotted and the point of sharp change was considered as the CMC (Figure 1C). 

*Scanning electron microscopy (SEM) and dynamic light scattering (DLS)*

To verify micelle formation and observe the morphologies of nanoparticles, scanning electron microscopy (SEM) was performed. Briefly, a concentration of 1 mg/ml of the PEG-di-fusion peptide copolymers in PBS (pH 7.4) was prepared, dropped on a carbon grid, and dried. Then samples were coated with gold and observed under an SEM electron microscope (MIRA3-TESCAN, Czech Republic). Micelles were diluted to 1 mg/ml in PBS (pH 7.4) in order to characterize their zeta potential, size distribution, and polydispersity index (PDI) by DLS using a Malvern Zetasizer Nano ZS (Malvern Instruments Ltd, Malvern, UK). To investigate the stability of the micellar nanovaccine, their size distribution, PDI, and zeta potential were traced on a 30 day period, both at 4 °C and 25 °C (Table 1). 

***Bone marrow derived dendritic cells (BMDC) separation and stimulation***

Dendritic cells were generated from hematopoietic progenitor cells of mice bone marrow according to our previous study (21). In brief, mice were sacrificed according to Mashhad University of Medical Science ethical committee protocols. The fresh bone marrows of the femur and tibia were extracted and their monocytes were washed twice with RPMI medium. Progenitor cells were seeded and cultured *ex vivo* at a density of 1.0 × 10^6^ cells/ml in IMDM media (Invitrogen) supplemented with 10% heat-inactivated FBS and 1% penicillin/streptomycin for 6 days at 37 °C and 5% CO_2_ incubator. Stem cells were differentiated into immature DCs by adding 5 ng/ml IL-4 and 25 ng/ml GM-CSF (Gibco, USA). 

*The ability of nanovaccine in induction of BMDC maturation*

**Figure 2:**
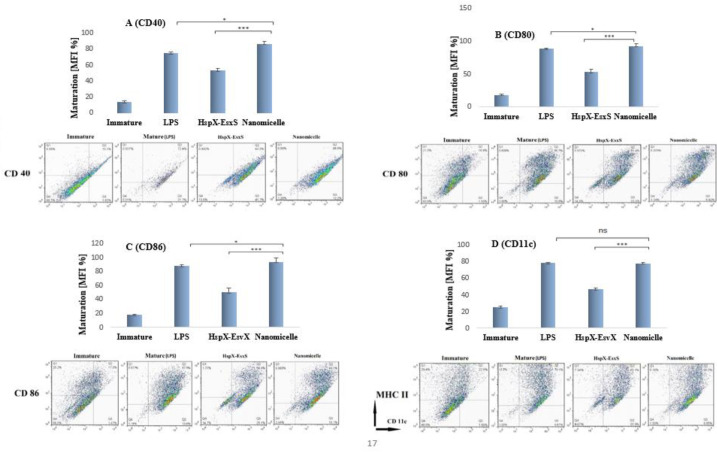
Graphs (A) to (D) represent the impact of HspX-EsxS and micellar nanovaccine (PEG - HspX-EsxS) on the maturation of dendritic cells (DCs) using flow cytometry analysis. Dot plots show the percentage of positive dendritic cells for surface markers. Ns means non-significance (*P*-value> 0.05), * means *P*-value≤0.05, ** means *P*-value≤0.01, and *** means *P*-value≤0.001, and data is reported as mean ± SD (n = 3)

On day 7, DCs were incubated with micellar nanovaccine, di-fusion protein, and LPS in order to investigate the effect of formulations on DC maturation. Immature DCs were incubated with micellar nanovaccine (9.25 mmol/ml), fusion protein (2.5 μg/ml), and LPS (100 ng/ml, Invivogen) in separate cell culture dishes. After 8 hr, cells were washed thrice with specific wash buffer containing 1% BSA and 2% FCS and incubated for 30 min with a mixture of diluted tagged specific CD marker antibodies including, FITC-CD11c, PE-CD40, PE-CD80, PE-CD86, and MHC-II (BD Biosciences) in the dark at 4 °C. The expression of maturation markers was quantified by flow cytometry (FACS Calibur flow cytometer, BD Biosciences), and data were analyzed using FlowJo analysis software (20) (Check Figure 2).

***Immunization studies***

To verify whether the *in vitro* results of DCs activation and stimulation are interdependent with the *in vivo* immunogenicity results, an immunization study was performed. Female 6-week old BALB/c mice were purchased from Pasteur Institute of Iran (Tehran, Iran) and kept under specified conditions in the animal house of Mashhad University of Medical Science. All experiments were carried out under the standardized guidelines confirmed by the Animal Ethics Committee of the Mashhad University of Medical Sciences (#87639). Based on previous studies (15), the micellar nanovaccine and control groups were administered three times with a 2-week interval, subcutaneously or nasally, according to the immunization schedule shown in Table 2. 

***Cytokine assay***

*Cell culture*

Three weeks after the last immunization, mice were sacrificed. Spleens were removed and placed into a 2 ml cold incomplete RPMI medium and vortexed severely with a bead-beater (Biospec, OK) to obtain single cells. The splenocytes were transferred into a cold RPMI medium and centrifuged at 300 g at 4 °C for 5 min. The supernatants were aspirated and cell pellet dispersed in 10 ml cold ammonium chloride (pH 7.4) as lysis buffer, for 5 min to lyse the remaining red blood cells and finally were washed again with complete RPMI. Pure cells were counted and seeded (3 × 10^5^ cells/5 ml) in 6-well cell culture plates in complete RPMI containing 10% FBS and 1% penicillin/streptomycin. Five µg/ml of the fusion antigen were added to the plates and incubated at 37 °C for 72 hr. Thereafter culture supernatants were collected in sterile microtubes and centrifuged at 3000 g for 2 min at 4 °C in order to remove any remaining cells (15). The final supernatants were collected and stored at -80 °C until the next assays.

*Determination of cytokines in supernatant of cultured cells*

**Figure 3:**
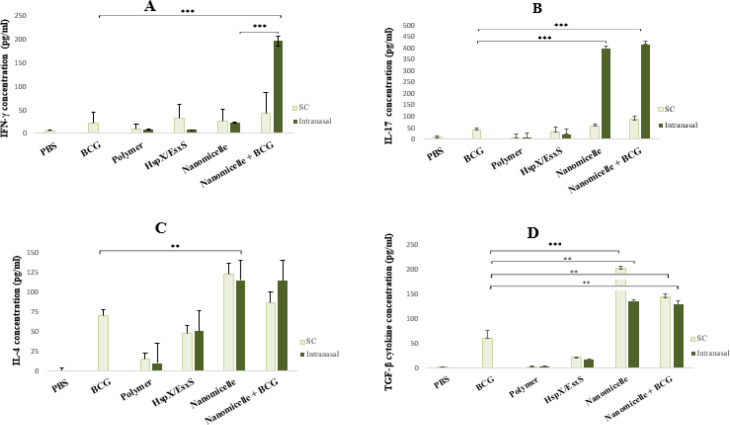
Level of concentration for cytokines IFN-γ (A), IL-17 (B), IL-4 (C), and TGF-β (D) in the supernatant of the re-stimulated splenocytes. The measurement is conducted using the sandwich ELISA method. Groups are compared with the BCG group. Groups with significant differences are labeled with a star. * means *P*-value≤0.05, ** means *P*-value≤0.01, and *** means *P*-value≤0.001, and data is reported as Mean ± SD (n = 6)

To assess the reflection of the nanomicellar formulation on the cell-mediated immunity, cytokine concentrations were measured in re-stimulated splenocytes cell culture medium. The amount of IFN-γ, IL-4, IL-17, and TGF-β concentrations in the cell culture supernatants were measured by an ELISA method using a commercial Kit (ParsToos, Iran), as previously described (15). Briefly, microtiter plates were coated with 1x capture antibody (100 µg⁄well) overnight at 4 °C. After blocking (2% BSA, for 1 hr at 4 °C), diluted solutions of the standards and samples from individual mice samples were applied into the wells and incubated for 1 hr at room temperature. Then detection antibody solution was added into wells and incubated for 1 hr at room temperature. In the next step, 100 µl of diluted streptavidin-HRP was added to each well and incubated for 1 hr at room temperature and detected with sure blue tetramethylbenzidine as substrate with a microplate reader Stat Fax 4200 (Awareness Technology, USA) at 450 nm with background subtraction at 630 nm. The plates were washed 5 times between steps with wash buffer (PBS pH 7.4, 10 mM + 0.05% Tween 20) (Figure 3). 

***Nasal lavage antibody titers***

**Figure 4:**
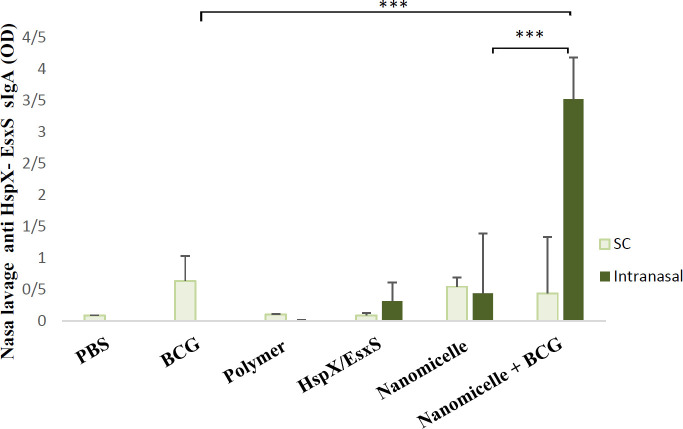
The OD of the anti HspX-EsxS sIgA antibody in different groups was measured. Groups are compared with the BCG group. Groups with significant differences are labeled with a star. * means *P*-value≤0.05, ** means *P*-value≤0.01, and *** means *P*-value≤0.001 and data is reported as Mean ± SD (n = 6)

The titers of antigen-specific antibodies (sIgA) in the nasal washes were determined by an ELISA method as described previously (15). Briefly, mice were sacrificed and their nostrils were washed with 1 ml PBS. 96-Well ELISA microplates were coated with 100 ng/100 µl of diluted HspX-EsxS di-fusion antigen in PBS buffer (0.05 M, pH 7.4) overnight at 4 °C. After blocking (2% BSA, for 1 hr at 4 °C), two-fold serial dilutions of lavage samples were applied to the plates. Diluted HRP-conjugated anti mice sIgA was added according to the kit’s instructions and detected by TMB (3,3′,5,5′-Tetramethylbenzidine, substrate for horseradish peroxidase). Washing was accomplished five times with wash buffer (PBS containing 0.05% Tween 20) between each incubation. The optical densities were measured on a microplate reader Stat Fax 4200 (Awareness Technology, USA) at 450 nm with background subtraction at 630 nm (Figure 4). 

***Statistical analysis***

All experiments in the experimental section were conducted three times, and data were expressed as mean ± standard error of the mean (SEM). One-way analysis of variance (ANOVA) followed by Tukey–Kramer post-test was used to compare the groups using SPSS (version 16). Differences regarded as statistically significant between the groups are presented as follows: * *P*<0.05, ** *P*<0.01, and *** *P*<0.001 (compared with the BCG group).

## Results

***Preparation and characterization of self-assembled nanostructures***

Synthesis of the polyethylene glycol-protein di-blocks (PEG-Protein) was accomplished by a direct EDC/NHS amide-bond between COOH of carboxy PEG and fusion protein-NH2 at 4 °C. FTIR and H-NMR techniques were used to confirm the formation of di-blocks. As shown in Figure 1A, in the protein-PEG spectrum, the highest peak is seen in ʋ = 3435 cm^-1^, while in the carboxy-PEG spectrum, the highest absorption band is seen in 3425 cm^-1^. This shift is due to the formation of amide bonds and the involvement of NH_2_ groups in the protein with the carboxyl group in carboxy-PEG, which reduces the percentage of intermolecular hydrogen bonding in the polymer. Besides this, in the protein spectrum, doublet absorption bands in the regions of 3440 and 3357 cm^-1^ were removed in the PEG-protein spectrum, which is another confirmation of the formation of the resulting amide bond connecting these two blocks. As shown in Figure 1B, in the carboxy-PEG spectrum, the peak seen in δ = 4.46-4.56 ppm, which corresponds to protons in the hydroxyl group related to the carboxyl group, has been removed in the PEG-protein spectrum. Besides this, in the protein spectrum, the peak seen in δ = 3.71 ppm which belongs to the protons of the amine group, has been removed in the ^1^HNMR spectrum of the PEG-Peptide structure, as a result of conjugation. This deletion is due to the formation of amide bonds and involvement of NH_2_ groups in the protein with the carboxyl group in carboxy-PEG. Other physicochemical properties of the nanoparticles such as size, shape, stability, and CMC were evaluated. Figure 1D shows two SEM images of the self-assembled nanomicelles with spherical shapes. As depicted in Table 1, the stability of intact nanomicelles was measured during 30 days at 4 and 25 °C, respectively. The present nanomicelles were 180–200 nm in size and showed good stability in terms of size, zeta potential, and PDI during 30 days of storage at 4 °C. However, when the study was performed at 25 °C, after the first week, the nanomicelles were not stable (Table 1). Measuring the CMC is the main way to check the formation and stability of the emerged micelles (28). We have calculated the CMC of the proposed nanomicelles as 0.024 mg/l (Figure 1C).

***Ability of nanovaccine in induction of BMDC maturation***

As shown in DC maturation results, nanomicelles could elicit more DC maturation compared with the di-peptide solution (*P*<0.001) or even LPS as positive control (*P*<0.01). As depicted in Figures 2A, 2B, and 2C, nanomicelles induced high maturation compared with LPS in terms of CD40, CD86, and CD80 marker expressions, respectively (*P*<0.05). Although the maturation indicated by the CD11c marker was not significant compared with other markers (Figure 2D), the maturation induced by nanomicelles was significantly higher than that induced by antigen solution in terms of the expression of all markers (*P*<0.01).

***Cytokine assay***

Figure 3 demonstrates the concentration of all cytokines after both nasal and subcutaneous administrations. While in the case of IFN- γ and IL-17 cytokines, as important Th-1 cytokines, there are no significant changes between mice SC immunized with the nanoparticles compared with controls (both HspX/EsxS solution and BCG); only in mice immunized nasally with the nanoparticles, IL-17 production is significantly more than controls (*P*<0.01). Notably, the concentrations of TGF-βand IL-4 as Th-2 cytokines in animals immunized with the nanovaccines were significantly higher than those of controls (HspX/EsxS solution and BCG groups), both for SC and nasal immunization. 

But, in the nasal and BCG + nanomicelle group, higher cytokine production is associated with Th-1 cytokines (both IFN- γ and IL-17 cytokines). As depicted in Figure 3, nasal administration of BCG followed by administration of nanomicelles, increased the concentrations of IFN- γ and IL-17 by 8.6 and 10 times, respectively, compared with BCG (*P*<0.001 for both cytokines) and nanomicelle alone (*P*<0.001 and *P*<0.05). But no further production of Th-2 cytokine was observed compared with exclusive nanomicelle administration. As shown in Figure 3, in BCG + nanomicelles group, production of Th-2 cytokines was similar to nanomicelles (*P*>0.05). 

***Nasal lavage antibody titers***

In order to evaluate the potential of different formulations in induction of the mucosal response, nasal sIgA titers were measured. Our nanomicellar form of antigen could elicit significantly higher mucosal (not systemic) responses as compared with antigen solution (*P*<0.001). As is demonstrated in Figure 4, the average mucosal immune response in BCG + nanomicelles is stronger than that reported for BCG (5.5 times) and nanovaccine alone (8.8 times). 

**Table 1:** **D**ata represent the averaged-hydrodynamic diameters (nm), zeta potential (mv), and the PDI of the nanomicelles at 4 °C and 25 °C; in both conditions, the nanomicelles were dispersed in PBS buffer (pH 7.4). Data was reported as mean ± SD (n = 3)

		**0 day**	**7 day**	**15 day**	**30 day**	
4 °C	Hydrodynamic diameters (nm)	210.6	240.8	238.33	247.2	
	Zeta potential (mv)	-24.03	-26.4	-25.7	-27.4	
	PDI	0.403	0.472	0.480	0.496	
25 °C	Hydrodynamic diameters (nm)	210.6	286.03	383.8	540.7	
	Zeta potential (mv)	-24.03	-24.2	-25.96	-28.06	
	PDI	0.403	0.383	0.45 0.591		

**Table 2:** Groups of immunized mice and the associated quantities

Administration groups	Injection quantity
PBS buffer (pH; 7.4, 10 mM) (PBS)	100 μl (subcutaneous), 5 μl (each nosestrill)
Commercial BCG vaccine (BCG)	5×10^6^ CFU/mouse
Polyethylene glycol solution (Polymer)	Equal polymer content per dose (2.2 μg)
HspX/EsxS protein (Antigen)	10 μg/dose
Nanomicelles composed from protein-polyethylene glycol di-block (Nanomicelles)	Contained 10 μg fusion protein/dose
Prime BCG followed by boost nanomicelles (BCG+nanomicelles)	(5×10^6^ CFU + 10 μg antigen)/mouse

CFU: colony-forming unit

## Discussion

The clinical use of live or whole-cell vaccines has limitations due to their safety issues. The recombinant antigenic peptides are safe but weak immunogens. In the studies on TB vaccines, to partly overcome these limitations, multistage subunit vaccines composed of a few recombinant proteins linked together have been constructed (22, 23). These antigens are expressed in different cell cycles of tuberculosis bacteria and could induce immune responses by different mechanisms (24). A number of TB antigens have recently been used as potential TB vaccine candidates, two of them are HspX and EsxS antigens. Combination of TB antigens from early antigenic proteins plus latency-associated antigens is a new approach in vaccine design (16, 25-27). 

These fusion proteins are safer and provide several antigenic epitopes, however, they mostly provide poor immunogenicity, and require an immunoadjuvant to increase their immunogenicity potential. Several approaches have been explored to improve the effectiveness of fusion proteins as TB or other vaccines and nanoparticulate adjuvants are among the most studied ones (28).

In the present study, we have linked a HspX/EsxS di-fusion protein to a hydrophilic molecule (polyethylene glycol), and the resulting di-block polymer could self-assemble to the nanomicelles. This nanomicelle could act as a type of particulate antigen delivery system/adjuvant that better presents the loaded antigen to the antigen-presenting cells (APCs). This better interaction leads to more potent responses (29). It is reported that these particulate systems exhibit very encouraging results in both systemic and nasal vaccinations (16). The formation of nanomicelles also was confirmed by electron microscopy and CMC evaluation (Figure 1). Due to the amphiphilic properties of the resultant di-block, the developed di-block was self-assembled into micelles by dissolving the di-block in distilled water. The stability and morphology of nanomicelle were checked and confirmed by DLS analysis and SEM imaging. 

Lower value of CMC indicates better and more stable nanomicelles upon dilution with biological fluids (30). It is worth noting that the micelles were administrated via nasal and SC routes, where low dilution occurs. Therefore, the low CMC values confirm that the nanomicelles remain intact and keep their original forms. Additionally, the high immune responses during DC maturation and cytokines assays, also confirm the stability of the nanomicelles upon dilution. 

A type of APCs that is present in the human body is dendritic cells (DCs). DCs are mainly available at the entrance of the antigen sites but they are also present in the blood and peripheral and lymphoid tissues (31). DCs will be matured by toll-like receptors when they face pathogen-associated molecules (32). When the DCs are well-matured, a cascade of immune system stimulation will be triggered (21). To test the efficiency of prepared nanovaccine in terms of DC maturation, four main maturation markers were evaluated *ex vivo. *The results are presented in Figure 2, where the expression of each marker is separately sketched and compared with the positive control group (LPS). Induction of DC maturation by nanomicelles represented similar results evidenced with different maturation markers. Overall, the *ex vivo* DC maturation results confirmed the efficacy of nanomicelles. These data confirm the fact that the particulate form of the antigen shows more uptake than the soluble antigens (33). 

To study the immunoadjuvant effect of the nanomicelles, the designed nanovaccine was parenterally and nasally administrated. Moreover, to evaluate the possible synergistic effect of the proposed nanovaccine, a prime-boost state (BCG followed by nanomicelles administration, exhibited as BCG+nanomicelle in Figures) was tested both after parenteral and nasal administrations. Four cytokines related to Th-1 response (INF- γ and Th-17) or Th-2 response (IL-4 and TGF-β) were studied.

IFN-𝛾 is the main cytokine in the Th-1 profile and enhances phagosome maturation, activation of oxidative mechanism in macrophages, and activates autophagy mechanism, to control and stop the infection (34). Th-17 as a major CD+4 T cell subtype produced IL-17 as pro-inflammatory cytokines. The protective role of IL-17 in tuberculosis has been confirmed in many studies (35, 36). Some studies demonstrated elevated IL-17 is required to enhance the recruitment of IFN-𝛾 producing cells in the lung. IL-17 mediates the induction of granuloma formation and also could mediate other independent IFN-𝛾 mechanisms of protection against tuberculosis (37). Then the concentration of the selected cytokines was measured in cultured splenocytes collected from 10 groups of mice. According to our results, the present subunit vaccines induce cellular arm of immune response rather than humoral response (check Figure 4). As mentioned in the results section, more cytokine secretion was observed in the Th-1 profile, and more specifically in nasal administration of the nanomicelle+ BCG group (more detail in Figure 4). The relatively high ratio of IFN-γ/ IL-4 (1.7), which revealed Th1-type immune response, was observed in mice immunized nasally by BCG + nanomicelles. Among primed BCG groups, this pattern was repeated in nasally immunized mice and showed the highest ratio of IL-17 / TGF-β among all vaccinated groups. In fact, addition of a prime BCG into the nasal vaccine regimen induced a strong shift in the IL-17 / TGF-β ratio. Overall, the balance between IFN-γ and IL-17 is likely to affect the disease outcome or the designed vaccine performance (36, 38). Researchers have also shown induction of higher IL-17 concentrations in nasally immunized mice in previous studies (20). It has also been reported that high IL-17 concentration could induce and prolong the mucosal immunity and memory T cell responses, a finding that does seem to depend on the stage of *Mtb* infection (20). 

It seems that the efficiency of new subunit vaccines in potentiating the effect of the BCG vaccine could be varied depending on several factors. It may be varied by the type of antigen, the life cycle of the bacterium, the time period the antigen belongs to, vaccinated animal or human, administration of vaccine before or after infection, subunit vaccine considered as therapeutic or prophylactic vaccine, type of nanoparticle used, and antigen encapsulation in nanoparticles or surface-linked to them (39-42).

Besides the key role of cellular immunity in protection against TB, mucosal immunity is inevitable. Specifically, since the majority of TB infections enter via the mucosal route, mucosal immunity plays an important role in prevention and treatment. BCG as the only licensed prophylactic TB vaccine is administered intradermal which induces systemic immune responses accompanied by very low mucosal immune responses. A practical approach to compensate for the weakness of BCG-induced mucosal immunity is to use an additional nanoadjuvanted vaccine that stimulates mucosal responses. It is reported that in nasal immunizations, mucosa-associated lymphoid tissue can uptake particulate antigens more easily than the soluble antigens, resulting in higher mucosal and systemic responses (43). sIgA is a secretory predominant antibody isotype that widely exists in the mucus layers and plays a critical role in the mucosal immune system. sIgA maintains the homeostasis between commensal microorganisms and pathogens by entrapment of the incoming pathogens in the mucus (44). Elevated levels of antibodies in the nasal mucosa indicate a positive immune response to the nasal immunization. The same results were observed in our study. 

Nanomicelles could elicit significantly higher mucosal (not systemic) responses as compared with the antigen solution (*P*<0.001). BCG+nanomicelle also stimulates more mucosal immune response than that reported for BCG (5.5 times) and nanovaccine alone (8.8 times) (check Figure 4).

There is s no clear data on the mechanism of the mucosal immunity against *Mtb*. The designed nanomicelles act more efficiently when administered nasally, as compared with SC administration. This difference could be attributed to the stealth nature and also mucoadhesion properties related to exposed PEG chains on the surface of the nanomicelles. It is previously shown that PEGylated nanoparticles strongly promote mucosal delivery, presumably by interpenetration of PEG chains in the mucus matrix. Additionally, forming hydrogen bonds between the oxygen atoms in PEG chains and the mucin glycoproteins could directly accelerate the mucosal delivery. As a result, the nanoparticles will attach to mucus for a longer time, leading to a higher chance of uptake by the resident APCs. However, when a PEGylated nanoparticle (stealth nanoparticle) is applied subcutaneously, it is well concealed from APCs and therefore it can escape better than the naked nanoparticles. So it is rational to achieve better results in nasal administration, either with or without prime BCG (45, 46).

## Conclusion

It is difficult to find a new and efficient alternative for the current BCG vaccine. In an effort to improve the *Mtb* vaccine, a di-fusion antigen of TB was connected to PEG and resulting di-block co-polymer with the ability to self-assemble to nanomicelles. The micellar nanovaccine was able to elicit good DC maturation. Nanomicelles could efficiently induce systemic cytokines as well as nasal sIgA titers. The expression pattern of cytokines indicated the superiority of cellular immunity. Nasal administration of two doses of nanomicelles after a prime subcutaneous administration of BCG created the highest mucosal and systemic immune responses. 

## Authors’ Contributions

ZF Conceived the project, helped carry out investigations, performing the experiments, data curation, formal analysis, software analysis, writing the original draft, and critical revision and editing of the article. MRJ Supervised the work. MS Processed the data and supervised the work. SZ Performed experiments. MT Helped with funding acquisition, supervision, project administration, formal analysis, and final approval of the version to be published.

## Conflicts of Interest

The authors declare that they have no conflicts of interest relevant to this article.
